# First record of *Euphlyctis kalasgramensis* (Anura: Dicroglossidae) from Punjab, Pakistan

**DOI:** 10.1080/23802359.2020.1731337

**Published:** 2020-02-28

**Authors:** Waqas Ali, Arshad Javid, Ali Hussain, Muhammad Hafeez-ur-Rehman, Anne-Lise Chabber, Farid Hemmatzadeh

**Affiliations:** a Department of Wildlife and Ecology, University of Veterinary and Animal Sciences, Lahore, Pakistan;; b Department of Fisheries and Aquaculture, University of Veterinary and Animal Sciences, Lahore, Pakistan;; c School of Animal and Veterinary Sciences, The University of Adelaide, Adelaide, Australia

**Keywords:** *Euphlyctis kalasgramensis*, *Euphlyctis cyanophlyctis*, 16s RNA

## Abstract

The present study documented the first record of *Euphlyctis kalasgramensis* from Punjab, Pakistan. The specimens were collected during field visits from June through August 2018. Various morphometric measurements of *E. kalasgramensis* were taken and compared with *Euphlyctis cyanophlyctis.* Snout-vent length (SVL) was 38.11 ± 0.87 mm (*n* = 5), snout length was 3% of SVL, foot length was 55% of SVL, head length was 32% of SVL and weight was 8.01 ± 0.12 g (*n* = 5). A few specimens (*n* = 2) were euthanized and preserved for molecular analysis through mitochondrial 16S rRNA gene sequences. The newly obtained DNA sequences of *E. kalasgramensis* were submitted to GenBank and accession numbers were obtained (MK881165.1 and MK920114.1). The Maximum likelihood and Neighbor-joining trees based on Kimura 2-parameter distance resulted in similar phylogenetic trees. *Euphlyctis kalasgramensis* was out group in both phylogenetic trees. The interspecific divergence of *E. kalasgramensis* and *E. cyanophlyctis* was high ranging from 4% to 6% as compared to low intraspecific divergence 0% and 1%. The diversity and distribution ranges of many amphibians species are not well known in Pakistan due to lack of taxonomic information. In our recommendation, a large scale DNA barcoding is required to report more cryptic or new species from Pakistan.

## Introduction

Geographically Pakistan is represented by Palearctic and Oriental regions; lies at latitude 24°, 37° N and longitude 61°, 78° E. The country can be divided into 15 habitat types in three major divisions; mountainous ranges, foothills, and Indus plains. The climate is continental type having considerable variations during winter and summer temperatures. The monsoon season ranges from July to September and rainfall varies throughout the year (Ali et al. 2018).

Globally, 7481 amphibians species have been reported so far representing three major groups, namely Caudata, Anura, and Gymnophiona. The arid to semi-arid climatic conditions makes Pakistan less favorable for amphibians to thrive and only 24 species belong to four families, namely Bufonidae, Megophryidae, Microhylidae, and Ranidae, have been reported (Khan 2006, 2011). Amphibians are distributed from sea level to 4000 meters in the Himalayas ranges in Pakistan and stretching from longitudinal 60° 52′ to 75° 22′ E to latitudinal 24° to 37° N (Ali et al. 2018).

Identification of amphibians on morphological parameters is still considered reliable however there is ambiguity in the taxonomic information of many amphibians species in Pakistan. There is a need to apply modern molecular techniques for species identification through mitochondrial DNA genes sequencing. In this context, the present study documented the first record of *Euphlyctis kalasgramensis*, a cryptic species to its sister taxon *E. cyanophlyctis* from Punjab, Pakistan.

## Materials and methods

### Sample collection and preservation

Specimens were collected from selected sites of Punjab, Pakistan during field visits. Only two specimens euthanized and preserved in 70% alcohol for molecular analysis. The voucher specimens (Voucher numbers: ZMUVAS1 and ZMUVAS5) were deposited at Zoological Museum, Department of Wildlife and Ecology, University of Veterinary and Animal Sciences, Pakistan.

### Morphological measurements

Measurements were taken with digital calipers to the nearest 0.02 mm. Morphological measurements were taken following the definitions of Ali et al. (2016) includes snout to vent length (SVL), snout length (SL), eye diameter (ED), horizontal tympanum diameter (HTYD), vertical tympanum diameter (VTYD), head length (HL), hand length (HAL), foot length (FTL), and weight (W).

### DNA extraction and sequencing

Total genomic DNA was extracted from preserved liver and muscle tissues (less than 50 mg) by DNeasy tissue kits (Qiagen, Switzerland) as per the manufacturer’s instructions. Quality of DNA was checked through agarose gel electrophoresis and concentration was measured using Thermo scientific NanoDrop One. DNA samples were brought to PC-2 lab at School of Animal and Veterinary Sciences, The University of Adelaide, Australia for DNA barcoding.

mtDNA fragment 16S rRNA was amplified using 16SA-L (5′-CGCCTGTTTATCAAAAACAT-3′) and 16SB-H (5′-CCGGTCTGAACTCAGATCACGT-3′) primer set (Vences et al. 2005). PCR amplification was carried out in 25 µl volume reaction with 2–5 µl of DNA. The PCR amplification comprised of 94 °C for 3 min; 40 cycles at 94 °C for 30 s, 55 °C for 30 s and 72 °C for 1 min; and a final 10 min at 72 °C. The PCR products were checked on 1.2% agarose gels. Purification of PCR products was performed using the Qiagen purification kit and all the samples were sequenced in both directions using dideoxy chain termination direct Sanger sequencing on AB3730xl sequencer (AGRF, Australia) according to standard protocols.

### Data analysis

DNA sequences ambiguities were edited in Bioedit software 7.2, forward and reverse reads aligned using Clustal X (Tamura et al. 2011; Kumar et al. 2012). Obtained DNA sequences were submitted to GenBank and accession numbers were obtained. The closely related sequences of *E. kalasgramensis* and *E. cyanophlyctis* retrieved from GenBank for phylogenetic tree analysis. The phylogenetic analyses were performed using Maximum-likelihood (ML) and Neighbour-joining method with 100 bootstrap replicates using MEGA 6.0. Genetic distances within and between species were calculated using Mega 6.0 based on Kimura 2-parameters (K2P).

## Results

### Distribution

**Figure 1.**
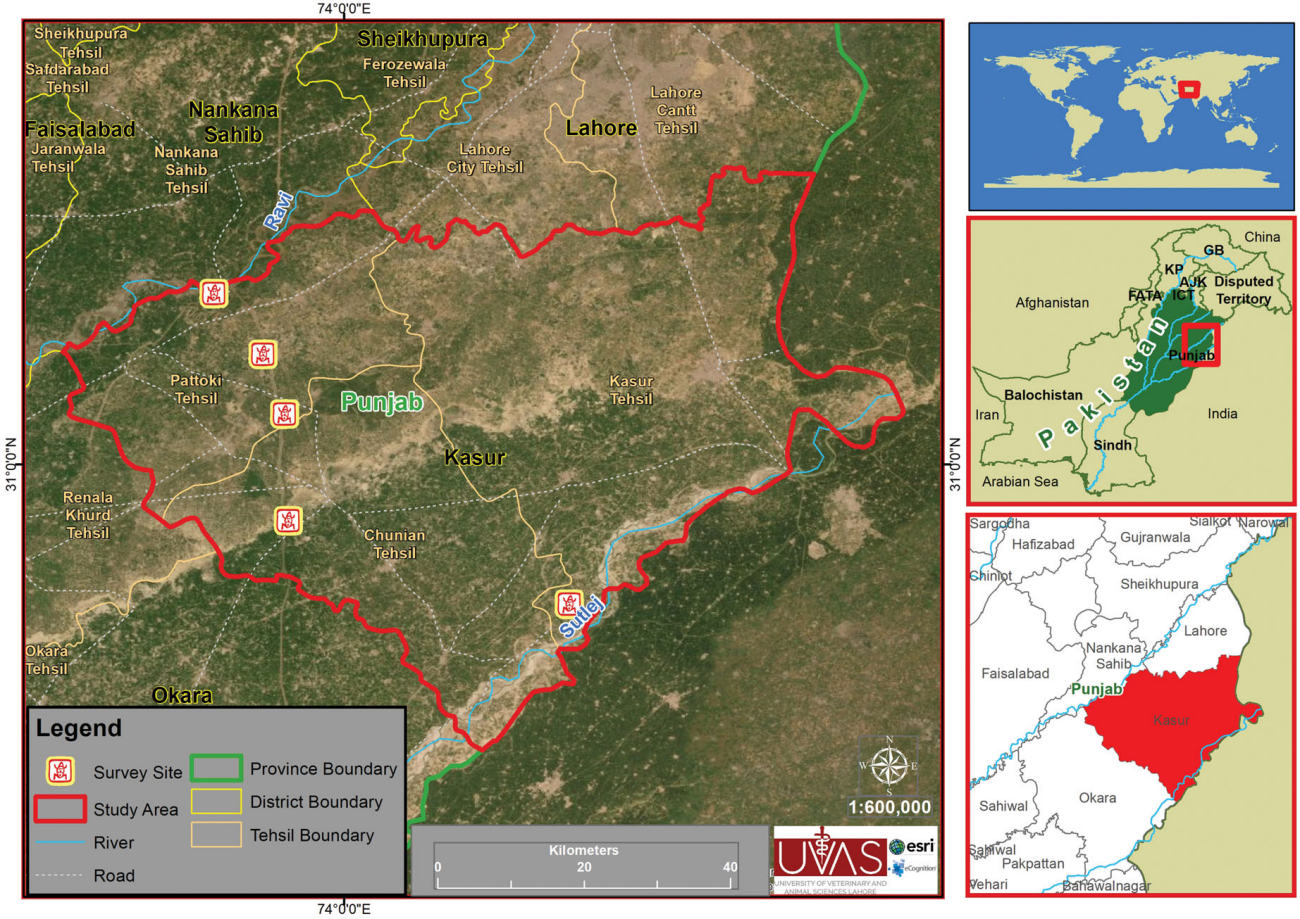
Distribution map of *Euphlyctis kalasgramensis* from Punjab Pakistan.

Specimens were collected during field visits from June through August 2018 from selected sites of Punjab province in Pakistan. Figure 1 shows the distribution map of *E. kalasgramensis* from the study area.

### Taxonomic position

Amphibia, Linnaeus, 1758

Anura Fischer von Waldheim, 1813

Dicroglossidae Anderson, 1871

*Euphlyctis* Fitzinger, 1843

*Euphlyctis kalasgramensis* Howlader, Nair, Gopalan, and Merilä, 2015

### Morphology

**Figure 2.**
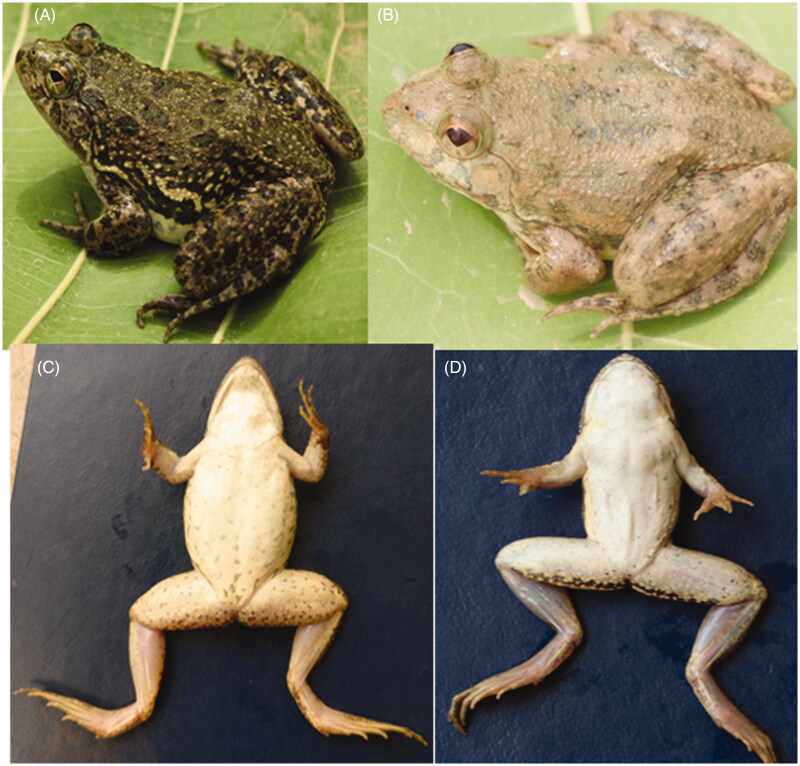
Diagnostic characters of genus *Euphlyctis.* Dorsal view of (A) *Euphlyctis cyanophlyctis* and (B) *Euphlyctis kalasgramensis*. Ventral view of (C) *Euphlyctis cyanophlyctis* and (D) *Euphlyctis kalasgramensis*.

*Euphlyctis kalasgramensis* (Figure 2) can be identified by following characters; small-sized frog, head is large and triangular as compared to *E. cyanophlyctis*, absence of mid-dorsal line, nostrils are round, small and much closer to snout tip. Snout is almost pointed, tympanum is round, fore limbs are robust, and fingers are small without webbing. Hind limbs are relatively longer than fore limbs and toes have well developed webbing. Dorsal surface is rough with warts, ventral surface is smooth, and adult females are larger than males. Snout-vent length (SVL) was 38.11 ± 0.87 mm (*n* = 5), snout length was 3% of SVL, foot length was 55% of SVL, head length was 32% of SVL, and weight was 8.01 ± 0.12g (*n* = 5). Morphometric measurements of *E. kalasgramensis* (*n* = 5) and *E. cyanophlyctis* (*n* = 5) are mentioned in Table 1.

**Table 1.** Comparison of mean body weight (g) and morphometric measurements (mm) of *Euphlyctis kalasgramensis* and *Euphlyctis cyanophlyctis* (Mean ± S.D).

Parameters	*Euphlyctis kalasgramensis* (*n* = 5)	*Euphlyctis cyanophlyctis* (*n* = 5)
SVL (mm)	38.11 ± 0.87	42.06 ± 1.11
SL (mm)	4.14 ± 0.98	3.25 ± 0.69
ED (mm)	3.17 ± 0.58	4.27 ± 0.44
HTYD (mm)	3.11 ± 0.10	4.10 ± 0.11
VTYD (mm)	3.12 ± 0.11	4.11 ± 0.21
HL (mm)	12.19 ± 0.99	17.10 ± 1.04
HAL (mm)	10.67 ± 1.11	12.15 ± 0.51
FTL (mm)	21.01 ± 0.19	22.12 ± 1.30
W (g)	8.01 ± 0.12	9.12 ± 1.62

SVL: Snout to vent length; SL: snout length; ED: eye diameter; HTYD: horizontal tympanum diameter; VTYD: vertical tympanum diameter; HL: head length; HAL: hand length; FTL: foot length; W: weight.

### Coloration

Dorsal surface is greenish-brown while ventral surface is white. However, in preserved form specimens become gray or grayish black and color pattern of the body faded with time as compared to live specimens.

### Natural history

*Euphlyctis kalasgramensis* found in water pools, channels, rice fields, and breeding season ranges from May through September. Male calls in group and courtship began as soon as female approaches preferred male. The male clung to the back of female and hold it with its forearms to form amplexus. The amplexed pair then moved to small, shallow water pool for spawning.

### Phylogenetic relationship

Previously, genus *Euphlyctis* is represented by *E. cyanophlyctis, E. cyanophlyctis microspinulata* and *E. cyanophlyctis seistanica* in Pakistan. mtDNA sequences of *E. cyanophlyctis microspinulata* and *E. cyanophlyctis seistanica* are not available in GenBank to validate their taxonomic position. The available 16S rRNA gene sequences of *E. kalasgramensis* (*n* = 5) and *E. cyanophlyctis* (*n* = 3) were retrieved from GenBank for phylogenetic trees analyses. The newly obtained DNA sequences of *E. kalasgramensis* were submitted to GenBank and accession numbers were obtained (MK881165.1 and MK920114.1). After trimming ambiguous bases, the obtained 16S rRNA fragments were 560 bp aligned with NCBI sequences comprised 530 bp.

**Figure 3.**
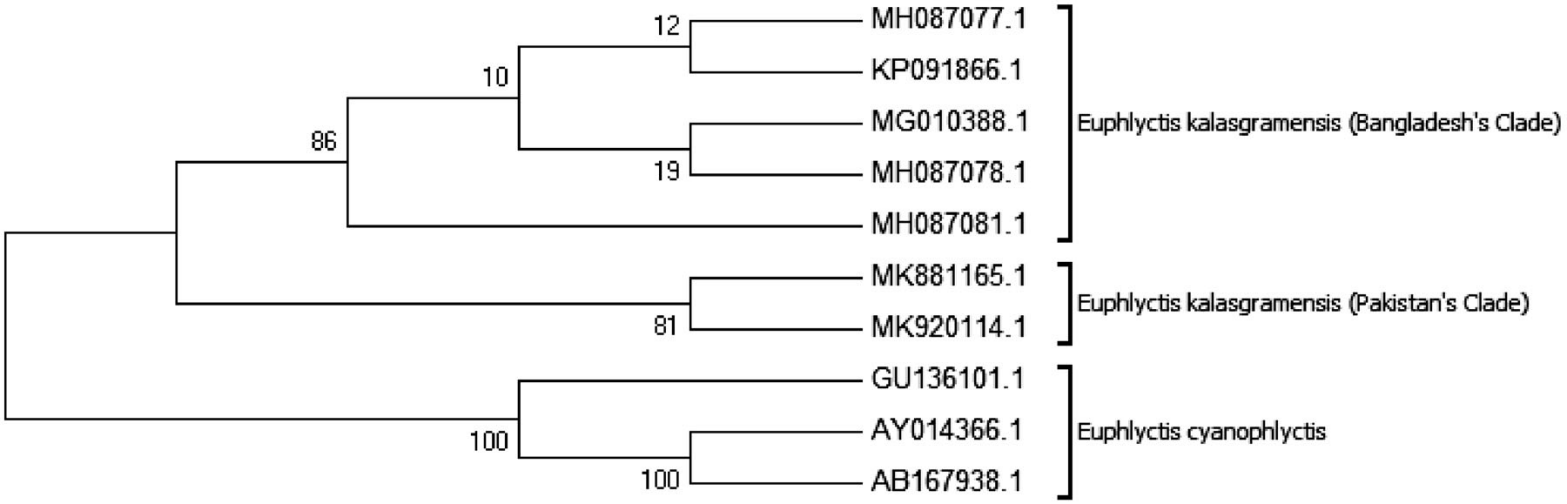
Phylogenetic relationships of *Euphlyctis kalasgramensis* and *Euphlyctis cyanophlyctis* using the maximum likelihood method based on the Kimura 2-parameter. Numbers on branches represent bootstrap values.

**Figure 4.**
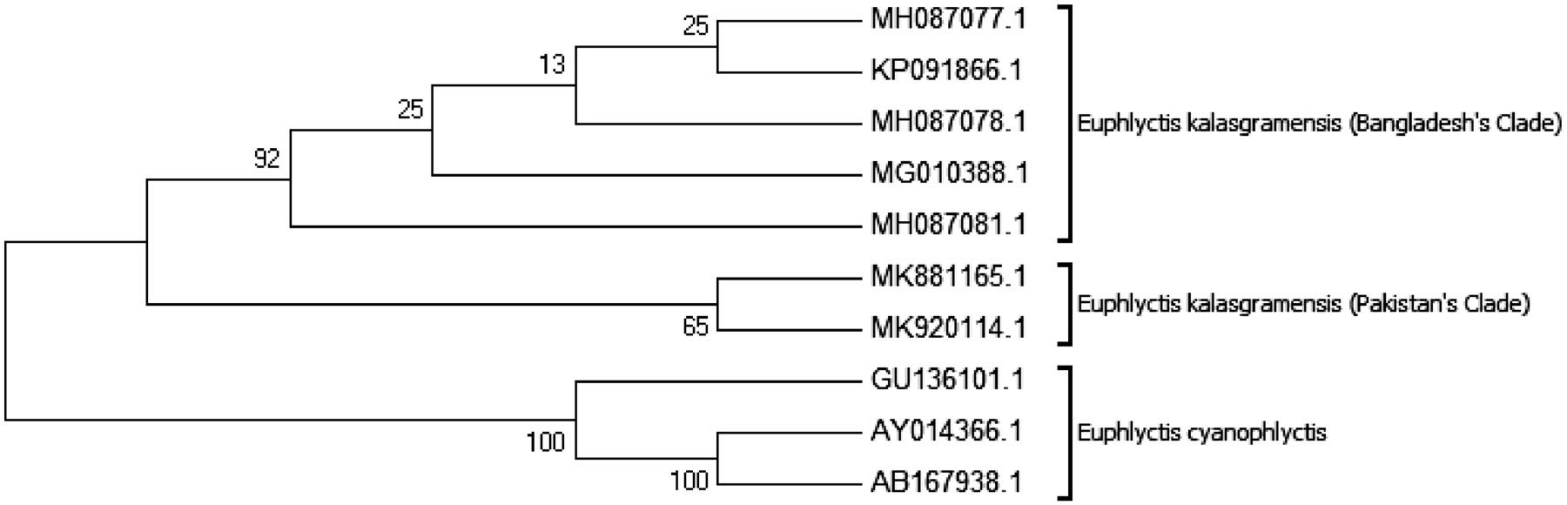
Phylogenetic relationships of *Euphlyctis kalasgramensis* and *Euphlyctis cyanophlyctis* using the neighbor-joining method based on the Kimura 2-parameter. Numbers on branches represent bootstrap values.

The Maximum-likelihood and Neighbour-joining trees based on Kimura 2-parameter distance resulted in similar phylogenetic trees (Figures 3 and 4). *Euphlyctis kalasgramensis* was out group in both Maximum-likelihood and Neighbour-joining trees. The interspecific divergence of *E. kalasgramensis* from *E. cyanophlyctis* was high ranging from 4% to 6% as compared to low intraspecific divergence 0% and 1% for 16S rRNA. Overall, 16S rRNA analysis showed an average of 3% interspecific variation between species of genus *Euphlyctis* (Table 2).

**Table 2.** The interspecific and intraspecific genetic identities of *Euphlyctis kalasgramensis* and *Euphlyctis cyanophlyctis* calculated using Kimura 2-parameter based on 16S rRNA gene.

Clade	*E. kalasgramensis*							*E. cyanophlyctis*		
	MK881165.1	MH087081.1	MH087077.1	MG010388.1	MH087078.1	KP091866.1	MK920114.1	AY014366.1	GU136101.1	AB167938.1
*E. kalasgramensis*										
MK881165.1	ID									
MH087081.1	0.993	ID								
MH087077.1	0.991	0.997	ID							
MG010388.1	0.993	1	0.997	ID						
MH087078.1	0.993	1	0.997	1	ID					
KP091866.1	0.993	1	0.997	1	1	ID				
MK920114.1	1	0.993	0.991	0.993	0.993	0.993	ID			
*E. cyanophlyctis*										
AY014366.1	0.952	0.95	0.948	0.95	0.95	0.95	0.952	ID		
GU136101.1	0.952	0.95	0.948	0.95	0.95	0.95	0.952	0.962	ID	
AB167938.1	0.948	0.946	0.944	0.946	0.946	0.946	0.948	0.991	0.958	ID

## Discussion

*Euphlyctis cyanophlyctis* is common species of Indo-Pak continent and shows close morphological similarities with other species of the genus *Euphlyctis* (Joshy et al. 2009). *Euphlyctis cyanophlyctis* was first documented by Schneider (1799) as *Rana cyanophlyetis* from eastern India. Many species that have been described within genus *Euphlyctis* complex are now grouped as synonymous species. In this context, *Rana bengalensis* (Gray 1830) and *Rana leschenaultii* (Cantor 1847) now considered identical to *E. cyanophlyctis*. These species have no comprehensive information on holotype availability and location (Frost 2014). *Rana bengalensis* and *R. leschenaultii* are similar to *E. cyanophlyctis* in having a spotted ventral part as compared to whitish ventral side of *E. kalasgramensis*. *Dicroglossus adolfi* described by Günther (1860) from Himalaya India considered same as Occidozyga and Bombina but later described as *E. cyanophlyctis* (Boulenger 1882).

Khan (1997) described *E. cyanophlyctis microspinulata* as 1st finger longer than 2nd and having microscopical spinules distributed on the body. However, *E. kalasgramensis* has equal first and second fingers and microscopical spinules are absent. De Silva (1958) reported *E. cyanophlyctis* as *Rana cyanophlictis* with two color varieties, namely ‘fulvus’ (Yellow) and flavens (green) body color. The description of these varieties does not match with *E. kalasgramensis* in terms of body coloration. Nikolskii reported *Rana cyanophlyctis* from Iran with close resemblance to Arabian *E. ehrenbergii* but later *E. ehrenbergii* also reported as same to *E. cyanophlyctis* (Dubois 1981).

Alam et al. (2008) conducted molecular analysis of all the known species in the genus *Euphlyctis* from Bangladesh. As a result, *E. mudigere* and *E. aloysii* were identified with their formal systematic description (Joshy et al. 2009). The molecular analysis of *E. kalasgramensis* matches with the genetic information of unnamed haplotype Ecya-Ba1 and Ecya-Ba2 reported by Alam et al. (2008) and later Howlader et al. (2015) provide detailed morphological description of the species.

The identification of cryptic taxa based on modern molecular techniques getting fame in Indo-Pak continent (Howlader 2010, 2011; Hasan, Islam, et al. 2012; Hasan, Kuramoto, et al. 2012). Previously, 24 amphibians species belong to nine genera, namely *Bufo*, *Scutiger*, *Microhyla*, *Uperodon*, *Euphlyctis*, *Fejervarya*, *Hoplobatrachus*, *Paa*, and *Sphaeroteca*, have been reported from Pakistan (Khan 2006). However, there is scanty of information on the taxonomy of many amphibians species in the country.

## Conclusions and recommendations

The diversity and distribution ranges of many amphibians species are not well known in Pakistan due to lack of taxonomic information. In this regard, a large scale DNA barcoding is required to report any cryptic or new species from Pakistan. Present study documents the first record of *E. kalasgramensis* from Punjab, Pakistan based on morphological and mtDNA 16S rRNA gene analyses.
